# Case Series: Cancer-Related Facial Pain Treated with Stellate Ganglion Block

**DOI:** 10.1089/pmr.2020.0042

**Published:** 2020-12-02

**Authors:** Raheleh Rahimi Darabad, Jerry P. Kalangara, Anna Woodbury

**Affiliations:** ^1^ Department of Anesthesiology and Pain Management, Indiana University School of Medicine, Indianapolis, Indiana, USA.; ^2^ Department of Anesthesiology and Pain Medicine, Emory University School of Medicine, Atlanta, Georgia, USA.; ^3^ Department of Anesthesiology and Pain Medicine, Atlanta VA Healthcare System, Atlanta, Georgia, USA.

**Keywords:** cancer, orofacial pain, stellate ganglion block, sympathetic ganglia

## Abstract

Stellate ganglion block (SGB) is believed to modify the pathologic sympathetic pain response and has been commonly used to treat complex regional pain syndrome. We report successful treatment of cancer-related facial pain with SGB in three patients, suggesting a possible sympathetic pain-related mechanism. All patients exhibited clinically significant improvement of pain 12 weeks following the procedure. SGB should be considered a palliative pain treatment option in cancer-related facial pain.

## Introduction

Stellate ganglion blocks (SGBs) have been used in pain medicine for almost a century.^1^ The block has traditionally been used to treat complex regional pain syndrome of the upper extremities,^2^ but has been applied for other indications as well. The block has also been shown to help reduce ischemic vascular spasms and arterial insufficiency in the upper limbs by diminishing sympathetic outflow and thereby increasing blood flow.^3^

SGB has also been shown to help with anxiety symptoms of post-traumatic stress disorder (PTSD) with effects lasting three to six months, possibly through its modulation of sympathetic output.^1,7^ It has been shown to have a statistically significant therapeutic impact on irritability or angry outbursts, difficulty concentrating, and sleep disturbance in patients with PTSD.^8^

SGB was reported to help with facial pain due to different etiologies, including burning mouth syndrome,^4^ and infectious processes, including Ramsey Hunt syndrome.^5^ Previous reports have indicated that SGB helps with orofacial cancer pain, with improvement of the ability to speak, chew, and swallow, as well as improvement of sleep.^6^ Oral squamous-cell carcinoma is one of the most common cancers, ranked sixth in the world, affecting ∼350,000 each year.^9^ Pain control is often multifactorial and refractory to treatment in patients with oral cancer, and symptoms can lead to malnutrition, limiting eating, drinking, and speaking for affected patients.^10^ Although SGB may be used for a variety of pain syndromes involving the upper extremities, head and neck, and although one case report has been previously published regarding the use of SGB and subsequent chemical ablation in orofacial cancer, we did not find previous cases utilizing nonablative SGB for therapeutic pain relief following chemotherapy and radiation. We report three patients suffering from cancer pain related to tumor or chemoradiation therapy who were successfully treated with SGB using local anesthetic.

## Case Series

Consent was obtained from the patients for the procedure, case report publication, and the use of the included picture. Three patients suffering from cancer-related facial pain were included in the case series. History, physical examination, and imaging findings were reviewed. Pain characteristics and previous treatments were reviewed. All patients underwent SGB under fluoroscopic guidance. Pre- and postprocedure and follow-up pain scores were recorded on the Numerical Rating Scale (NRS). Change of quality of life from baseline was assessed and recorded.

### Case 1

A 65-year-old male veteran with a history of tongue cancer stage IV with metastasis to the lungs, diagnosed 2 years prior, presented with pounding headaches, radiating to the eye, temporal, and frontal areas of the head, and associated throat pain with difficulty swallowing. He had undergone chemoradiation therapy to treat the malignancy, but now suffered from pain related to radiation burns around his neck. He had previously failed multiple nonsteroidal anti-inflammatory drugs (NSAIDS) (ibuprofen up to 800 mg po TID, not helpful), anticonvulsants (gabapentin titrated up to 900 mg po TID over the course of six months without sufficient relief, pregabalin 50 mg po TID attempted), antidepressants (venlafaxine 225 mg po daily without relief), opioids (hydrocodone po, oxycodone po, and fentanyl patches—all of which were somewhat helpful but weaned following accidental overdose), and topical therapies (lidocaine 5%, menthol/salicylate, and wintergreen oil, all of which provided some minimal benefit). He stated he was “tired of taking medications” and that he preferred nonpharmacologic therapies. He had not had any interventional procedures performed for his pain. He had a history of benzodiazepine and barbiturate use disorder but was not using these narcotics at initial evaluation in the pain clinic. He had a remote history of alcohol abuse and was a current smoker, but had a negative history of illicit drug use. Physical examination revealed no facial allodynia or hyperalgesia. He had pain with palpation of his forehead and mild tenderness to palpation of his upper throat, which was worse on the right side. He had an area of darkened skin and taut tissue related to radiation burns around his neck, worse on the right. The rest of the physical examination was unremarkable. An initial whole-body Positron Emission Tomography/computed tomography (CT) scan revealed uptake at the midline of the base of tongue and left lateral external tongue musculature, but no further findings suggestive of residual primary malignancy at the base of tongue after chemoradiation therapy.

### Case 2

An 87-year-old male veteran with a history of metastatic squamous-cell cancer (SCC) of the skull with ear involvement presented with left ear pain. It was burning in nature, sensitive to touch, and rated 10/10 at worst, 0/10 at best, and 8/10 on average. Pain was intermittent and triggered by touch and facial expression, lasting for hours. His primary complaint was an inability to taste food. He had failed to achieve relief with acetaminophen (syrup, 650 mg po q6hrs), opioids (fentanyl patch 50 μg/h, hydromorphone 4 mg po q3hrs, and hydromorphone 1.5 mg IV q2hrs PRN), and oral viscous lidocaine swishes (2%, 5 mL po QID). He had been refusing a prescribed gabapentin solution (250 mg po TID) due to difficulty swallowing. Physical examination revealed smoothing of the skin on the left side of the face, an erythematous bright-red left ear with active drainage, and a swollen left face. The rest of the physical examination was unremarkable. Head CT with and without contrast showed residual infiltrative soft tissue involving the external auditory canal, parotid gland/space, and masticator space.

### Case 3

A 65-year-old male veteran with a history of primary stage IVa SCC of the tongue presented with right tongue pain with radiation to the right face and ear. The pain was shooting and tingling. It was 10/10 at worst, 6/10 with ibuprofen, and 9.5/10 on average. The patient had experienced some benefit from acetaminophen (650 mg po q6hrs), NSAIDs (ibuprofen 800 mg po TID), and viscous lidocaine (2%, 5 mL po QID), but not complete relief. Gabapentin (300 mg po TID) was initiated with unclear benefit, per the patient. He was using a hydromorphone patient-controlled analgesic IV while inpatient, with inadequate control of his pain (using >60 mg morphine equivalents per day before the procedure). He was hesitant to take any medications and preferred procedures or surgery to treat the pain. Specifically, he stated he was afraid of the ibuprofen “burning a hole” in his stomach, and of the potential addictive and deadly side effects related to opioids. Physical examination was significant for a yellow-white right base of tongue lesion that was ulcerated. Neck soft tissue CT showed a large (measuring more than 6 cm in the greatest cross-sectional anteroposterior extent and at least 3.4 cm in the greatest cross-sectional transverse thickness), aggressive, deeply infiltrating, and heterogeneously enhancing right oral cavity mass, invading the floor of the mouth region, the right tongue, glossopharyngeal sulcus, and adjacent to the right lateral oropharynx.

### Procedure

**FIG. 1.**
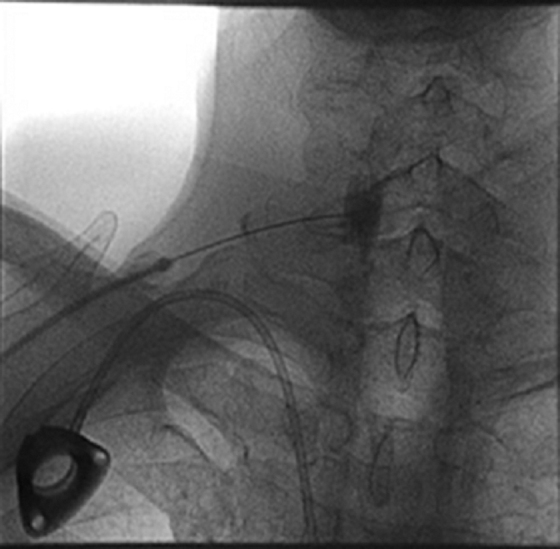
Fluoroscopic image of SGB in Case 1. Anteroposterior view following injection of radiocontrast dye confirmed appropriate spread at C6 Chassaignac's tubercle without vascular uptake. SGB, stellate ganglion block.

For each case, the block was performed with the patient in a supine position and in a sterile manner. Chassaignac's tubercle at C6 was identified using fluoroscopic guidance in an anteroposterior view. One milliliter of 1% lidocaine was injected subcutaneously at the site of entry using a 27G needle. Then, a 25G 3.5-inch needle was directed to Chassaignac's tubercle. Three milliliters of Omnipaque 180 mg/mL contrast dye was injected to ensure appropriate spread without vascular uptake. Then, 10 mL of 0.25% bupivacaine was injected, with negative aspiration every 2 to 3 mL, with no signs or symptoms of intravascular injection (Fig. 1).

## Results

Records were reviewed from various providers across the computerized patient record system, including palliative care practitioner notes, interventional pain clinic notes, and nursing assessments both inpatient and outpatient. Pain scores (NRS) were recorded by nursing staff as well as interventional pain practitioners immediately before and after each procedure, based on patient report. These were used for our assessment for immediate response, and long-term responses were obtained from palliative care and primary care provider notes as well as nursing intake assessments.

Patient demographics and medical history are summarized in Table 1. Pain characteristics are summarized in Table 2. All patients exhibited clinically significant long-term pain relief. Preprocedure pain scores 8, 10, and 5, improved to 4, 0, and 3, respectively, with a mean decrease in pain of 5.33 ± 4.16. Patients experienced between 40% to 100% pain relief for up to three months following the SGB. None of the patients had any unexpected side effects or complications as a result of the procedure.

**Table 1.** Patient Demographics and Medical History

	Age (years)	Sex	Type of cancer	Comorbidities	Cancer treatment
Case 1	65	Male	Stage IV tongue cancer	Depression, anxiety, GERD	Chemotherapy, radiation therapy
Case 2	87	Male	Metastatic SCC of the skull with ear involvement	HTN, heart failure s/p pacemaker	Palliative chemotherapy and radiation therapy
Case 3	65	Male	Stage IVa SCC of tongue	HTN	Palliative surgery

GERD, gastroesophageal reflux disease; HTN, hypertension; SCC, squamous-cell cancer.

**Table 2.** Characteristics of Pain in Three Patients

	Pain location	Pain description	Alleviating factors	Exacerbating factors	Associated symptoms	Failed medications	Pre-SGB NRS	Post-SGB NRS
Case 1	Throat, eyes, temporal and frontal head, anterior neck, TMJ	Burning	Magic mouth wash provided temporary benefit	Chewing	Difficulty swallowing	Advil, Aleve, Tylenol, gabapentin, hydrocodone, oxycodone, fentanyl patch, lidocaine topical, icy hot, wintergreen	8	4
Case 2	Left ear	Burning	—	Facial expression, touch	Difficulty swallowing	Tylenol liquid, fentanyl patch, gabapentin, hydromorphone, lidocaine viscous	10	0
Case 3	Right tongue, right face and ear	Shooting, tingling	—	Palpation	—	Ibuprofen, gabapentin, viscous lidocaine, Tylenol	5	3

NRS, Numerical Rating Scale; SGB, stellate ganglion block; TMJ, temporomandibular joint.

Case 1 experienced Horner's syndrome following the procedure, which caused him some concern, but was reassured that this was an expected effect of the procedure. He achieved ∼50% relief following the initial procedure while on stable medication dosing with pregabalin 50 mg po TID. Three months after the initial procedure, his pain had not yet returned to baseline. He received a follow-up SGB at three months that brought his pain down to 0, and did not require further analgesics for his cancer-related pain within a year following the second block. At that point, we began to address his noncancer-related arthritic low-back pain, which had become his primary concern.

Case 2 experienced ongoing relief three months following his procedure, had self-discontinued all opioids for pain control, and was on a maintenance dose of gabapentin 500 mg TID. He reported improved eating and activity, including gardening, eating more, driving, caring for his wife, and enjoying his life at home.

Case 3 was able to discontinue all opioid therapy for seven weeks following the procedure, although the pain did begin returning after seven weeks.

## Discussion

The stellate ganglion, also known as the cervicothoracic ganglion, is the fusion of the inferior cervical and first thoracic sympathetic ganglion. It is anatomically anterior to the neck of the first rib and seventh cervical vertebral body. It is a relatively large ganglion, measuring ∼2.5 cm in length, 1 cm in width, and 0.5 cm in thickness.^11^

The theory for the potential mechanism of cancer pain has been investigated in multiple studies. It is suggested that cancer-related oral pain is likely caused by stimulation of Ad and C fibers by mediators from cancer cells. These include a variety of mediators, including prostaglandins, bradykinin, chemokines, and other factors.^12^ One previous study showed that oral cancer pain was not correlated with mass size and suggested that pain could be related to nociceptive hypersensitivity or perineural infiltration.^13^ This case series, showing pain relief with SGB, suggests that sympathetically mediated pain is at least a component of cancer-related facial pain given this is a block of a sympathetic ganglion. Pain relief may be related to improved blood flow through blockade of the sympathetic pathway. Improvement of headaches with SGB may also be related to suppression of vascular wall edema.^3^

SGB for cancer-related facial pain might also help with pain through stress modulation. Some benefit with SGB has been documented in patients with PTSD, a chronic stress disorder caused by experiencing traumatic situations. Norepinephrine, associated with increased excitement and vigilance,^14^ is increased in the cerebrospinal fluid (CSF) of patients with PTSD.^15^ It is suggested that decreased sympathetic activity and norepinephrine (NE) level are the mechanisms of action of SGB for PTSD.

Despite the multiple potential benefits of SGB in patients with refractory upper extremity and facial pain, care must be taken in utilizing this therapy. Although none of our cases experienced complications related to the block, risks of the procedure include pneumothorax, intravascular injection, intrathecal injection, and nerve injury. SGB-related complications are typically a result of the proximity of the ganglion to vital structures. In one study of SGB, complications occurred in 33 out of 287 injections (11.1%); hoarseness/dysphagia was found to be the most common complication (54.6%) with local hematoma formation as the second-most common (33.3%), followed by ipsilateral arm numbness (6.1%), pneumothorax (3%), and contralateral Horner's syndrome (3%).^16^ It is important to note that ipsilateral Horner's syndrome is a known side effect and sign of successful sympathetic blockade of the stellate ganglion, typically resolving within a few hours following the block.^16^ In cases where Horner's syndrome is prolonged beyond the duration of action of the local anesthetic used for the block (2 hours for lidocaine or 8 hours for bupivacaine), other causes such as nerve injury or hematoma should be investigated. In our subset of patients, unilateral block ipsilateral to the side of greatest pain was performed to minimize the potential of bilateral injury or motor nerve blockade of the recurrent laryngeal nerve or vascular structures that rely on collateral circulation, which could lead to the need for intubation or resuscitation.

Image guidance is strongly recommended to reduce the risk of injury and unintentional intravascular or intrathecal injection. Use of CT^15^ and magnetic resonance imaging has been studied^16^ by different researchers but is not clinically practical. In this study, fluoroscopy was used, which is a common and routine approach for this procedure.^11^ Ultrasound is also commonly used for needle guidance in SGB, as it allows for visualization of nonbony structures such as the cervical, vertebral, and carotid arteries, as well as thyroid, esophagus, and nerve roots.^17^ In cancer patients, given the possible distortion of local anatomy related to orofacial cancer and scarring, careful evaluation of the injection site and feasibility of approach to the SGB utilizing preprocedure imaging should be performed.

Although all three of our subjects were treated with neuropathic pain medications before SGB, methadone was not attempted in any of them despite its N-methyl-D-aspartate antagonism properties. This was primarily related to patient preferences and the potential for adverse effects from methadone due to its long half-life and potential for QtC prolongation as well as delayed respiratory depression. A trial of methadone may be reasonable in select patients before pursuing SGB.

The main limitation of the current study is its small sample size and lack of a placebo control. Furthermore, we cannot assess our approach to the SGB and compare it with differing approaches with varying amounts and concentrations of local anesthetic or differing imaging modalities, such as ultrasound. However, current results from our three cases are promising and serve as a platform to produce pilot or larger studies to further investigate the efficacy of such a procedure for cancer-related facial pain.

We hope that sharing the results of this case series can improve treatment approaches and encourage the use of SGB for patients struggling with cancer-related facial pain before and after chemoradiation. However, further research is needed to make stronger recommendations, and the risks and benefits should be weighed for individual patients.

## Conclusion

SGB may be a viable option in the treatment of cancer-related facial pain both related to malignancy and to chemoradiation. Stress modification by means of SGB may have a role in the improvement of the psychological aspect of pain, which could potentially improve pain relief as well as quality of life in cancer patients.

## Abbreviations Used

CT: computed tomography
HTN: hypertension
NRS: Numerical Rating Scale
NSAID: nonsteroidal anit-inflammatory drugs
PTSD: post-traumatic stress disorder
SCC: squamous-cell cancer
SGB: stellate ganglion block
